# Where Do We Stand in the Domestic Dog (**Canis familiaris**) Positive-Emotion Assessment: A State-of-the-Art Review and Future Directions

**DOI:** 10.3389/fpsyg.2020.02131

**Published:** 2020-09-08

**Authors:** Erika Csoltova, Emira Mehinagic

**Affiliations:** Diana Pet Food, Elven, France

**Keywords:** behavior, dog, multiple measurement, physiology, positive-emotion

## Abstract

Although there have been a growing number of studies focusing on dog welfare, the research field concerning dog positive-emotion assessment remains mostly unexplored. This paper aims to provide a state-of-the-art review and summary of the scattered and disperse research on dog positive-emotion assessment. The review notably details the current advancement in dog positive-emotion research, what approaches, measures, methods, and techniques have been implemented so far in emotion perception, processing, and response assessment. Moreover, we propose possible future research directions for short-term emotion as well as longer-term emotional states assessment in dogs. The review ends by identifying and addressing some methodological limitations and by pointing out further methodological research needs.

## Introduction

There has been a growing interest in animal emotion research in recent years. However, positive-emotion has received much less attention than negative emotion thus far. Understanding how an animal perceives, processes, and expresses positive-emotion is crucial not only from a theoretical point of view to improve our knowledge about animal emotion, but also from a practical point of view to improve the animal’s life. Concepts such as satisfaction with life, well-being, and welfare refer to more than a mere absence of negative emotions linked to stress and suffering (Mench, 1998; Boissy et al., 2007; Mellor and Beausoleil, 2015; Lawrence et al., 2019); therefore, it is imperative to be able to objectively assess animals perception and processing of, and responses to, positive-emotions.

Specifically, this review focuses on positive-emotion perception, processing, and response assessment in dogs. The review starts with a brief overview of emotion, including its definition, functions of emotion, selected interdisciplinary approaches in the study of emotion, elements associated with and affecting emotion processing, and responses. It is not our intention to dive deeply into the wide and complex field of animal emotion, given the existence of rather extensive literature on the topic (e.g., Harding et al., 2004; Paul et al., 2005, 2020; Burgdorf and Panksepp, 2006; Boissy et al., 2007; Mendl et al., 2009, 2010; de Vere and Kuczaj, 2016; Massen et al., 2019; Kremer et al., 2020). Rather, we emphasize several important key aspects in the study of emotion. Next, we discuss positive assessment in dogs, including the definition of terms such as happiness and pleasure. Thereafter, we continue reviewing measures, approaches, methods, and techniques that have been implemented so far to identify positive-emotion perception, processing, and responses in dogs. Next, we propose possible future directions for research both in short-term emotional responses, as well as for longer-term emotional states assessment in dogs. The review ends by summarizing the main findings, identifying major methodological limitations, and suggesting possible solutions.

## A Brief Overview of Emotion

Even though there is ample literature on emotion ranging from psychology and neuroscience to animal welfare science, little agreement has been achieved on the concept of emotion. Conceptualization issues have been prevalent over the years and there has been a lot of debate about the definition of emotion. The ability to gain a better understanding of emotion and its complexity is complicated by several different factors. Major debates and disagreements have occurred, regarding the various components that make up emotions, how many emotions exist, the underlying structure of emotions, whether emotions require conscious experience, and how they should be measured (Daroff and Aminoff(eds), 2014; de Vere and Kuczaj, 2016). Furthermore, there are often discrepancies in how the authors define and use the terms across a given manuscript (de Vere and Kuczaj, 2016).

Although a widely accepted definition is missing, emotions are often described as relatively intense, rapid, affective responses to an external stimulus, causing a specific physiological change (Dantzer, 2002; Boissy et al., 2007).

One of the crucial roles of emotion from an evolutionary perspective is to facilitate behavioral and physiological adaptation to a changing environment. The essential survival function of emotions is to seek out rewards and resources while avoiding harm and punishment. The reward is associated with experiencing positive-emotion, while the consequence of reward omission or punishment is a negative emotional experience (Rolls, 2000, 2005).

Several theories have been proposed to explain emotion and emotion processing, both in humans and animals. Among them, is the discrete emotion theory, inspired by Darwin’s ideas on the existence of primary emotions reflected in a universal facial expression (Darwin, 1872; Tomkins, 1962; Ekman et al., 1987; Izard, 1994, 2007; Ekman and Cordaro, 2011) and affective neuroscience, which assumes a central role of specific hard-wired neural brain system responses in emotion processing (Berridge, 1996; LeDoux, 1998, 2000, 2012; Panksepp, 2004, 2005, 2011; Burgdorf and Panksepp, 2006; Damasio, 2011).

On the other hand, dimensional approaches, such as the theory of constructed emotion, consider emotions not as universal signals but as potential combinations of external and internal sensations evolved through various distributed brain circuits, which create subjective emotional experiences and perceptions (Barrett, 2006, 2017; Barrett and Wager, 2006). The theory assumes the universal existence of a fundamental conscious neurophysiological affective state called core affect consisting of two dimensions, vertical degree of activation (arousal) and horizontal degree of pleasure (valence) (Russell and Barrett, 1999; Russell, 2003; Barrett, 2006; Duncan and Barrett, 2007).

To bring the discrete and dimensional approaches together, Mendl et al. (2010) have proposed an overarching integrative and functional psychobiological approach for the study of animal emotion. This framework offers a structure for different discrete emotions integration and suggests causal bidirectional interaction between short-term discrete emotions and longer-term mood states. It emphasizes the strong influence of emotional responses to environmental situational factors on an individual’s emotional mood and related behavioral, physiological, and cognitive changes. The integrative functional framework also proposes how to measure and predict the impact of emotion-induced cognitive biases on mood states and how these mood states, in turn, impact the stimulus appraisal and decision-making processes (Mendl et al., 2010).

Apart from taking into consideration the mood state when studying animal emotion, here we would like to stress the possible importance of temperament traits in animal emotion assessment studies. Temperament has been considered as a consistent and long-lasting disposition to qualitatively affect both the mood state and the susceptibility to emotional stimuli, speed, and strength of emotion responses (Allport, 1961). Temperament has been defined by certain researchers as a biogenetically determined, relatively stable emotional predisposition to process and express emotions—one that serves as a foundation for personality^1^ (Goldsmith et al., 1987). Given that, it is plausible to assume a significant role of certain temperament traits in animal emotion perception, processing, and responses (Boissy, 1995; Boissy et al., 2007; Coleman, 2020; Kremer et al., 2020).

For a long time, it was believed that emotion is independent of cognition (Zajonc, 1980). However, recent neurobiology research has offered proof about the interconnected relationship between emotion and cognition (Okon-Singer et al., 2015).

Besides, research on animals provides further evidence for interactions between emotion and cognitive processes such as learning, memory, attention, judgment, decision making, and social cognition (Harding et al., 2004; Paul et al., 2005; Boissy et al., 2007; Mendl et al., 2009, 2010; Burman et al., 2011; Burman and Mendl, 2018; Hintze et al., 2018; Massen et al., 2019). Hence, examining cognitive processes may prove particularly valuable when studying dogs’ positive-emotion processing and responses.

It has been suggested that emotion contributes to cognitive processing, while cognition can play a role in the regulation of emotion (Izard et al., 1984, 2008; Harding et al., 2004; Mendl et al., 2009). For instance, a cognitive appraisal is an assessment that is made about the relevance of a stimulus to oneself at a particular point in time (Lazarus, 1966). The appraisal needs to be made to assign emotional value to stimuli by making use of available information, to determine the appropriate response (e.g., to fear, flee, or feel pleasure). How the animal appraises the information is going to affect the valence and arousal of the emotional response (Paul et al., 2005). However, emotional factors may interfere with correct information processing and create so-called cognitive biases. Cognitive bias is a pattern of a systematic error in cognitive processing by creating a subjective representation of reality that differs from an objective input (Haselton et al., 2015). It is influenced by the emotional state and directly impacts attention, judgment, and decision making. Therefore, cognitive bias can be a useful indicator of emotion in animals (Harding et al., 2004; Mendl et al., 2009, 2010; Bateson et al., 2011; Roelofs et al., 2016; Burman and Mendl, 2018; Clegg, 2018).

To sum up, emotions are short-term reactive responses to emotion-eliciting stimuli (potentially rewarding or punishing) accompanied by physiological changes in the body (Dantzer, 2002; Rolls, 2005; Boissy et al., 2007). They have biological and cognitive foundations. Emotions consist of either positive or negative valence and arousal, ranging from low to high (Russell and Barrett, 1999; Mendl et al., 2010). From a temporal perspective, emotions are closely associated with longer-term mood states and long-lasting, relatively stable temperament traits. Mood, temperament, and cognition seem to play significant roles in emotion processing and responses (Goldsmith and Campos, 1982; Gross(ed.), 2007; Mendl et al., 2009, 2010).

## Positive-Emotion Assessment in Dogs

Assessment of positive-emotions in dogs has received only limited attention thus far. Research studies have been focused mainly on negative emotion assessment (e.g., stress, fear, and anxiety) (Dreschel and Granger, 2005; Bergamasco et al., 2010; Travain et al., 2015; Csoltova et al., 2017). One of the possible reasons for this bias is that indicators of negative emotions are much more intense and therefore more easily observed and studied. Positive-emotional states associated with an animal’s well-being are usually much more subtle, often less expressive, and often difficult to reliably assess and distinguish from negative emotional states (Boissy et al., 2007). Even though dog well-being and welfare^2^ topics have received increased attention in recent years, most of the studies have focused on indicators of compromised dogs’ welfare (e.g., Beerda et al., 2000; Rooney et al., 2007; Mariti et al., 2015) or indicators related to the improvement of dogs’ already compromised welfare (Hennessy et al., 1998, 2006; Coppola et al., 2006; Bergamasco et al., 2010; Shiverdecker et al., 2013; Csoltova et al., 2017).

Understanding emotions, particularly positive-emotions, is crucial for dogs’ well-being. Therefore, there is a huge need for studies focused on the positive well-being and welfare of dogs. Concepts of positive well-being, welfare, and quality of life are closely linked to positive-emotions associated with concepts such as happiness and pleasure (Duncan, 2005; Boissy et al., 2007; Yeates and Main, 2008; Lawrence et al., 2019).

Happiness can be defined as an emotional state that is characterized as a longer-lasting, steady, persistent, and yet less intense positive-emotional experience (Boissy et al., 2007). Animal happiness might include everyday experiences of pleasure, opportunities to interact with their environment, conspecifics, caretakers, and having the freedom to achieve one’s own goals (Yeates and Main, 2008).

**FIGURE 1:**
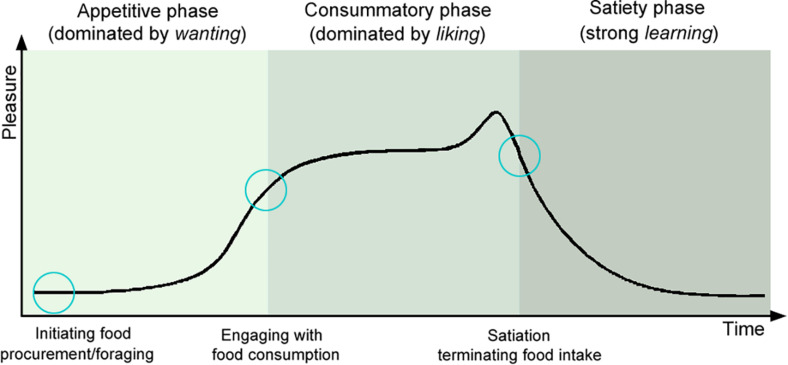
Pleasure cycle phases (adapted from Berridge and Kringelbach, 2011; Rømer Thomsen et al., 2015). Pleasures associated with different social and non-social rewards, follow a cyclical pattern, represented by appetitive, consummatory, and satiety phases (Sherrington, 1907; Craig, 1917). Rewards play a crucial role in initiating, sustaining, and switching between the phases and consist of processes supported by multiple brain networks, each with a specific function in the processing of *wanting*, *liking*, and *learning* elements of the reward (Berridge, 1996; Berridge and Kringelbach, 2013).

To the best of our knowledge, pleasure has been the most thoroughly researched positive-emotion so far (e.g., Cabanac, 1971, 1979; Panksepp, 1982; Berridge, 1996; Berridge and Kringelbach, 2008, 2011). Pleasure, although rather a complex phenomenon, is usually associated with subjective hedonic experience (Berridge and Kringelbach, 2011). Pleasure is a passive experience, evoked by an anticipated or received reward, which affects learning, approach behavior, and decision making, as well as possibly contributing to a longer-lasting state of happiness (Schultz, 2015). Pleasure is intertwined with reward-related processes such as *wanting*, *liking*, and *learning* (Robinson and Berridge, 1993; Berridge and Kringelbach, 2008; Berridge et al., 2009). These motivational, emotional, and cognitive processes alternate and co-appear at any time during the pleasure cycle and can be both conscious and unconscious (Berridge, 1996; Berridge and Robinson, 2003; Finlayson et al., 2007; Berridge and Kringelbach, 2008; Berridge et al., 2009). The pleasure cycle (Sherrington, 1907; Craig, 1917; Robinson and Berridge, 1993; Berridge and Kringelbach, 2011) consists of the following time phases (Figure 1):

1. The appetitive phase is characterized by *wanting*, motivational processes of incentive salience, desire to obtain the reward (Berridge, 1996; Berridge and Robinson, 1998; Berridge et al., 2009; Kringelbach and Berridge, 2009). *Wanting* is mainly triggered by innate unconditioned or classically conditioned stimuli (Robinson and Berridge, 1993; Berridge et al., 2009). Dopamine seems to be a reward predictor, reflecting *wanting* (Berridge and Robinson, 1998; Wise, 2004; Robinson et al., 2005; Arias-Carrión and Pöppel, 2007; Berridge, 2007; Newquist and Gardner, 2015). This phase is characterized by increased activity, motivation, approach behavior, and associative learning (e.g., remembering food-associated stimuli) (Craig, 1917; Berridge, 1996; Berridge et al., 2009; Schultz, 2015).
2. The consummatory phase is characterized by *liking*. During this stage, the subject experiences sensory pleasure (Berridge, 1996; Berridge and Kringelbach, 2008; Berridge et al., 2009). Opioids, endocannabinoid, and orexin seem to mediate the hedonic *liking* responses to food reward (Peciña and Berridge, 2005; Smith and Berridge, 2005; Mahler et al., 2007; Ho and Berridge, 2013; Castro and Berridge, 2014).
3. The satiety phase is characterized by strong *learning* about reward predictions based on previous experiences (although learning happens at any phase during the reward cycle). Learning includes explicit cognitive predictions as well as implicit classical and operant conditioning (Kringelbach and Berridge, 2009). Leptin, ghrelin, and serotonin have been implicated, among others, in satiation and satiety (de Graaf et al., 2004; Voigt and Fink, 2015; Cassidy and Tong, 2017). It has been suggested that social rewards are processed at the same reward network as nonsocial rewards and addiction (Wang and Aragona, 2004; Young and Wang, 2004; Krach et al., 2010; Liu et al., 2010).

In the following subsections, we are going to review in more detail what methodological and technical advancements have been achieved and what positive-emotion measures have been identified so far in dogs’ positive-emotion perception, processing, and response assessment. We start with behavioral measures, including research on brain lateralization of emotion processing, facial expression analysis, and qualitative approaches. Thereafter, we discuss specific noninvasive physiological and endocrine measures applied in positive-emotion assessment. Finally, we discuss neurobiological methods applied in dog emotion research.

### Behavioral Measures

Behavioral signals play an important role in intra- and interspecies emotional communication. Expressing emotions through facial and body movements has been documented in several studies (Beerda et al., 1997, 1998; Rehn and Keeling, 2011; Shiverdecker et al., 2013). However, some of the behavioral indicators mentioned below have been associated with both negative and positive-emotions.

Thus far, research has focused mainly on identifying behaviors associated with negatively connotated emotions. Although there exists a high within-individual variability in behavior expression, behaviors such as increased activity, repetitive movements, auto-grooming, lowered body posture, lip licking, panting, yawning, crouching, shaking, vocalization (barking, whining), scratching, paw lifting, paw sweating, increased salivation, blinking of eyes have been identified as stress indicating behaviors in dogs (Hetts et al., 1992; Beerda et al., 1997, 1998, 1999; Dreschel and Granger, 2005; Tod et al., 2005; Hennessy et al., 2006; Siniscalchi et al., 2008; Stracke et al., 2011; Hekman et al., 2012; Shiverdecker et al., 2013; Kuhne et al., 2014b; Csoltova et al., 2017).

On the other hand, increased physical activity (Rehn and Keeling, 2011; McGowan et al., 2014), attentive behavior (Rehn and Keeling, 2011; Westerback, 2011), tail wagging (Rehn and Keeling, 2011; Takahashi, 2011; McGowan et al., 2014), lip licking (Rehn and Keeling, 2011; Westerback, 2011; Gygax et al., 2015), vocalization, and shake off (Rehn and Keeling, 2011) have been associated with positive-emotional states in dogs.

#### Vocalization

Dogs use a wide range of different context-specific subunits of barks and mixed sounds as a means for communication of emotional arousal and both positive and negative emotional states. However, high variability has been found among, as anywhere from 2 to 12 types of subunit barks were identified in different breeds (Feddersen-Petersen, 2000), consisting of acoustic parameters such as frequency, tonality, and rhythmicity (Pongrácz et al., 2010).

Apart from intraspecies communication, research also shows that dogs’ barking represents an important active communication tool also with humans (Feddersen-Petersen, 2000; Yin, 2002; Pongrácz et al., 2005, 2006). In addition, humans seem to be able to correctly identify the emotional state of the dog based on context-specific barks. Short inter-bark time lapses are perceived as aggressive, while longer time lapses between barks indicate low aggression. Higher pitched barks combined with longer between bark sequences are associated with happiness and playfulness (Yin and McCowan, 2004; Pongrácz et al., 2005, 2006). However, excessive barking has been linked to the excitement, boredom, disturbances, anxiety, and pain in dogs (Righetti, 2005).

Whining and yelping sounds were recorded when the owner returned after separation, suggesting increased positive arousal and motivation to approach the owner (Rehn et al., 2014). Another study found that early onset of whining after short separation from the owner was the most typical vocal response of dogs diagnosed with a separation-related disorder. According to the authors of the study, barking encompasses a wide range of both positive and negative emotional states, while, on the other hand, whines are more associated with negative emotions (Pongrácz et al., 2017).

#### Activity

Activity, in a form of exploration or information gathering, might be a relevant indicator of the animal’s well-being (Boissy et al., 2007). Increased activity was shown as a sign of positive excitement in dogs when reunited with the owner after separation (Rehn et al., 2014) or when the dogs were solving a cognitive task (McGowan et al., 2014). On the other hand, this behavioral indicator is also context specific, since excitation was also documented as an indicator of moderate stress in a social setting (Beerda et al., 1998, 2000) and shelter environment (Part et al., 2014; Cozzi et al., 2016).

However, activity measured as speed or latency to approach food in subsequent runway tests might be a relatively easy and effective tool to measure incentive salience in dogs. The operant runway method has been proved to be a useful approach to study the behavioral and neurobiological basis of reward-seeking motivation in rodents (Hull, 1934; Crespi, 1942; Ettenberg, 2009). Research suggests that the time necessary to cross the runway is a reliable indicator of the animal’s incentive salience (Ettenberg, 2009). The runway method may, therefore, be an interesting alternative for exploring food-related behavior in dogs.

Recently, two studies implemented the runway task as a tool to test its usefulness in food preference in dogs. Both research groups found that dogs moved faster through the runway to obtain a more preferred food compared to the less preferred food (Riemer et al., 2018; Cameron et al., 2019). However, the food quantity did not affect the running speed, suggesting a higher incentive value of the more preferred food over a greater quantity of less preferred food (Riemer et al., 2018).

#### Lip Licking

Observed higher frequencies of oral behavior in a form of lip/nose licking and/or tongue flicking have previously been proposed to indicate acute stress in a social context (e.g., Beerda et al., 1997, 1998; Csoltova et al., 2017). Nose licking has been also observed in frustration-provoking situations when access to a food reward was denied to dogs (Bremhorst et al., 2019).

Albuquerque et al. (2018) studied dogs’ lip licking responses to both positive and negative stimuli. They have found more prevalent frequency of lip licking in response to negative visual stimuli (human’s and dogs’ angry and aggressive facial expressions) compared to positive ones (humans’ and dogs’ happy and playful expressions). In addition, authors have found higher rates of lip licking in response to human stimuli.

On the other hand, other research findings suggest that lip licking could be associated with positive-emotion, performed in response to verbal and physical human interactions and seeing an owner after separation (Rehn and Keeling, 2011; Westerback, 2011; Gygax et al., 2015).

#### Tail Wagging

A higher frequency of tail wagging recorded in social settings has been proposed as an indicator of emotion reflecting positive valence and contact-seeking behavior (Rehn and Keeling, 2011; Rehn et al., 2014). Tail wagging related to positive expectation was observed during treat offerings (Travain et al., 2016). Also, dogs were observed to wag their tails more as a reaction toward food reward or human contact and less to conspecifics (McGowan et al., 2014).

#### Play Behavior

Play behavior is a pleasurable experience and thus a possible indicator of a positive-emotional state. Play behavior includes a complex set of different motor activities and play patterns. Through play, dogs acquire specific skills necessary for survival (Bekoff, 1974a). Social play has an important role in developing social experiences and skills (Bekoff, 1974b, 1984). The high prevalence of social play in adult dogs indicates its social cohesion and bonding function (Bradshaw et al., 2015; Palagi et al., 2015; Sommerville et al., 2017). In addition, it was found that the affiliative behavior of the human handler during play decreased cortisol levels in working dogs (Horváth et al., 2008).

However, there have been some debates regarding whether play behavior is a reliable indicator of positive welfare since the play occurs under less favorable environmental conditions as well (Held and Špinka, 2011; Sommerville et al., 2017).

#### Behavioral Lateralization of Emotion Processing

Extensive evidence exists about brain lateralization in perception and response to emotion-eliciting stimuli in different animal species (e.g., Fernández-Carriba et al., 2002; De Boyer Des Roches et al., 2008; Rogers, 2010; Wallez and Vauclair, 2011; Leliveld et al., 2013; Rogers et al., 2013; Siniscalchi et al., 2016c). The main existing brain lateralization theories propose different explanations of emotional processing. The right hemisphere hypothesis states that the right cerebral hemisphere dominates the perception and regulation of emotional processes independent of the emotion valence (Borod et al., 1998). On the other hand, the valence-specific hypothesis suggests that each cerebral hemisphere is responsible for different emotional valence perception and processing, with the left hemisphere being dominant in positively connotated emotion processing, while the right hemisphere predominantly processes negative emotions, such as fear and aggression (Ahern and Schwartz, 1979; Wedding and Stalans, 1985; Adolphs et al., 2001).

In the following subsections, we review research done on behavioral lateralization of emotion processing, including emotional information perception and emotional expression.

##### Visual lateralization

Emotional facial expressions communicate one’s desires and intentions and may influence others’ emotional state. Therefore, they play an important role in social interactions. The animal’s ability to accurately recognize and discriminate emotional information, including the facial emotions of others, is a core element of social competence promoting adaptive behavior in response to perceived information (Lindell, 2013).

Research evidence exists that dogs are capable of facial recognition (Racca et al., 2010; Somppi et al., 2014). In addition, further studies revealed pronounced specificities regarding facial processing, demonstrating dogs’ ability to discriminate between different emotional facial expressions in humans (Nagasawa et al., 2011; Müller et al., 2015; Turcsán et al., 2015; Somppi et al., 2016). Besides, dogs looked significantly longer at both human and dog faces whose facial expression reflected the same emotional valence as the vocalization. These results suggest that dogs’ brains process, differentiate, and integrate multimodal sensory inputs of different emotional valence (Albuquerque et al., 2016).

Apart from dogs’ ability to recognize individual faces visually and discriminate among different emotional states, further studies also revealed gaze bias and lateralized cerebral hemispheric processing of facial expressions. Strong left-gaze bias was recorded in dogs toward images of human faces, but not toward images of monkeys, dogs, and inanimate objects (Guo et al., 2009). Further study revealed that dogs looked significantly more into the right side of the face, regardless of the emotion expressed in their left visual field, indicating the right cerebral hemispheric dominance for processing all emotions. Facial expressions depicting positive-emotion resulted in forehead gaze fixation, while gaze fixation on the eye and mouth region was recorded in response to negative facial expression (Barber et al., 2016).

Within the framework of the valence-specific hypothesis, left-gaze bias was observed in dogs in response to pictures depicting negative facial expressions of conspecifics and a right-gaze bias in response to positive expressions. No gaze bias was observed in response to neutral dog facial expressions. In response to pictures depicting human facial expressions, the right cerebral dominance (left-gaze bias) was found in response to negative and neutral expressions; however, no gaze bias was recorded toward positive expressions (Racca et al., 2012).

In a different study, human facial expressions depicting the same emotion (happiness, surprise, disgust, fear, sadness, anger, or neutral) were simultaneously presented into the left and right visual fields of the dog during feeding. Faces depicting anger, fear, and happiness resulted in left-turn bias, while surprise facial expression resulted in right head-turn bias. Shorter head-turning reaction time was recorded for fear and anger facial expressions in comparison with other emotional stimuli. For the facial expression depicting disgust and the neutral facial expression, no head-turning bias was observed. Further, angry human facial expressions resulted in longer latency to resume eating compared to other emotional facial expressions. Increased stress-associated behavioral responses to angry and happy human facial expressions were observed. Lastly, fearful, angry, and happy emotional expressions resulted in significantly higher cardiac activity in dogs, indicating dogs’ sensitivity to human emotional facial expression (Siniscalchi et al., 2018).

Apart from studies on dogs’ lateralized emotional facial-expression perception, lateralized head-orienting responses were observed to other visual emotion-eliciting stimuli in dogs as well. Silhouettes depicting a dog, a cat, or a snake were simultaneously presented into the left and right visual hemifields of the dog while feeding. Cat and snake pictures resulted in a left head-turning bias and shorter reaction time, suggesting right hemispheric activation for threatening and alarming stimuli. No head-turning bias was observed for a silhouette of the dog. When stimuli were presented either to the left or right visual spaces, dogs were found to react more to the left than to the right-side presentations, regardless of the stimulus presented. Furthermore, both left head-turning bias and the presentation of a cat and a snake silhouettes into the left visual space required a longer latency to resume feeding (Siniscalchi et al., 2010).

##### Acoustic lateralization

It seems that dogs are able to differentiate between positive (laughing) and negative (crying) emotional auditory stimuli (Huber et al., 2017), and additional research has revealed contra-lateralized brain processing in response to human emotional vocalization (Siniscalchi et al., 2008). In particular, the analysis of human nonverbal vocalizations showed a clear left head-orienting toward negative emotional stimuli (fear and sadness), suggesting a prevalent activation of the right cerebral hemisphere. On the contrary, a clear right head-turning response was recorded in response to positive vocalization (happiness), suggesting dominant left cerebral hemisphere activation in dogs. Moreover, heart rate and behavioral responses have provided further confirmatory evidence that human emotional sounds induced emotional responses in dogs (Siniscalchi et al., 2008).

##### Olfaction lateralization

Contrary to other senses, olfactory information projects predominantly to the ipsilateral hemisphere, meaning that right nostril sensory input is processed mainly in the right cerebral hemisphere, while the left cerebral hemisphere processes mainly the input from the left nostril (Royet, 2004). Previous research on dogs’ olfactory behavior has found nostril bias when investigating and processing odors (Siniscalchi et al., 2011, 2016b; Brown and Reimchen, 2020). Novel and non-aversive stimuli resulted in an initial right nostril sniffing preference, followed by a switch to left nostril investigation during repeated exposures. Consistent right-nostril sniffing bias was observed for potentially arousing emotional stimuli such as the sweat of a veterinarian and adrenaline (Siniscalchi et al., 2011). The right nostril bias, in response to novel and arousal stimuli, suggests sympathetic activation, which is predominantly controlled by the right cerebral hemisphere (Craig, 2005).

Asymmetric nostril use was also observed in response to human and canine odors produced during emotional states such as joy, fear and anxiety, physical stress, and neutral stimuli. Right nostril bias was observed when dogs investigated conspecific stress-indicating odors (collected when the dog was isolated in an unfamiliar environment). Left nostril bias was documented when dogs sniffed human odors produced during fear and physical stress (running). These results, according to the authors, indicate that dogs might use different sensory pathways for conspecific versus heterospecific emotional signals processing (Siniscalchi et al., 2016b).

##### Facial expression lateralization

A study examining facial expression lateralization in dogs in response to positive social stimuli (the owner) found that dogs moved their left eyebrow more after reuniting with the owner compared to the baseline. No facial expression bias was recorded for positive nonsocial stimuli (such as a dog toy). The authors have concluded that the left eyebrow bias most probably reflects dogs’ attachment to the owner (Nagasawa et al., 2013).

##### Motor lateralization

Rapidly growing empirical research suggests evidence also for motor lateralization in dogs. Researchers have found a link between the paw and visuospatial preference. Left-pawed dogs were found to consume more food kibbles on the left side of the feeding apparatus and similarly, right-pawed dogs ate more of the food on the right side of the feeding apparatus. Ambidextrous dogs did not show any bias. This evidence indicates an association between motor laterality and visuospatial bias in dogs, similar to the one found in humans (Siniscalchi et al., 2016a).

Interesting findings have been reported from a study investigating the association between motor laterality, emotional state, and cognitive bias in dogs. The emotional state was assessed by a cognitive test assessing dogs’ food-approach latency. The food bowl was positioned in one of three ambiguous positions. According to the study, the distributions of lateralized (57%) and non-lateralized (43%) dogs were roughly equal. It was found that left-pawed dogs tended to be more negative in their cognitive processing than right-pawed or ambidextrous conspecifics. The study proposes that paw preference, as an indicator of hemispheric dominance, can reliably predict cognitive bias in dogs and can therefore represent a quick and useful prevention tool to identify animals that are at risk from negative welfare, thus enabling quick interventions to improve their well-being (Wells et al., 2017).

Research studies are suggesting that bilateral asymmetry of tail-wagging is also related to the valence of emotional stimuli (Quaranta et al., 2007; Artelle et al., 2011; Siniscalchi et al., 2013). Differential amplitudes of lateral asymmetry of tail wagging were found in response to the type of emotion-eliciting visual stimuli the dogs were presented with. Dogs presented with stimuli eliciting approach motivation (seeing dog’s owner) performed higher amplitude of right-side tail wagging. By contrast, stimuli associated with withdrawal motivation (dominant unfamiliar dog presentation) resulted in a higher amplitude of left-side tail wagging (Quaranta et al., 2007).

According to the findings, dogs not only respond with asymmetric tail wagging to qualitatively different emotional stimuli (Quaranta et al., 2007) but are also sensitive to the asymmetric tail wagging displayed by conspecifics (Artelle et al., 2011). Higher cardiac activity and anxiety-indicating behavior were recorded when dogs watched left-side tail wagging compared to right-side tail wagging video recordings of conspecifics (Siniscalchi et al., 2013).

### Facial Expression Analysis

A facial expression is nonverbal communication that has both survival and adaptive function (Erickson and Schulkin, 2003). Most nonhuman animals produce an automatic involuntary display of facial expressions in response to specific emotion-eliciting stimuli. Lately, there has been an increase in the number of studies on the facial expression of pain in different animal species (Langford et al., 2010; Sotocina et al., 2011; Keating et al., 2012; Costa et al., 2014; Holden et al., 2014; Guesgen et al., 2016; McLennan et al., 2016; Finka et al., 2019). Further, it has been proposed that facial expression may reliably indicate both negative and positive-emotional states and thus have a substantial potential in animal welfare assessment (Descovich et al., 2017).

One of the methods used, to noninvasively measure human muscle movements is called the Facial Action Coding System—FACS (Ekman and Rosenberg(eds), 2005). This research tool enables the user to manually code almost any anatomically possible facial expression. To identify and code facial movements in nonhuman species, AnimalFACS was developed and adapted from the original human FACS. This coding system facilitates intra-species and inter-species comparisons of facial expressions (Vick et al., 2007; Parr et al., 2010; Waller B. M. et al., 2012; Caeiro et al., 2013, 2017a; Micheletta et al., 2015; Wathan et al., 2015).

Research has documented that domesticated dogs have developed facial muscles, enabling facial expression of emotion, unlike their wolf ancestors (Waller B. M. et al., 2013; Kaminski et al., 2019). DogFACS has been used to study dog facial expressions in response to different emotional stimuli and compare them with human facial expressions (Waller B. et al., 2013). Emotional stimuli eliciting happiness (such as initiation of play with the owner), positive anticipation (related to food and outdoor activity), frustration (inaccessible toy, food, or space), and fear (experience of a thunderstorm or visualization of specific objects) were used. Researchers identified distinctive stimuli-dependent facial expressions in dogs, although these expressions differed from human facial expressions in response to categorically similar emotional stimuli (Caeiro et al., 2017b).

Research groups studying hedonic and aversive reactions toward taste stimuli found homologous inter-species facial expressions. The results revealed similar distinctive facial expressions in response to sweet and bitter taste stimuli, as well as in the intensity of the emotional reaction (Grill and Norgren, 1978; Berridge et al., 1981; Berridge, 2000; Steiner et al., 2001). Thus far, dogs have not been observed to display specific facial expressions in response to palatable food; however, an increase in “ears adductor” has been observed during anticipation of food reward (Bremhorst et al., 2018).

### Qualitative Behavioral Assessment

In recent years, qualitative methods to assess animal emotion and welfare have been gaining more and more attention (e.g.,Wemelsfelder et al., 2000; Wemelsfelder and Farish, 2004; Knierim and Winckler, 2009; Walker et al., 2010; Stockman et al., 2011; Rutherford et al., 2012; Fleming et al., 2013; Wemelsfelder and Mullan, 2014; Konok et al., 2015b).

Qualitative Behavioral Assessment (QBA) is a holistic, noninvasive, positive, and dynamic method using fixed lists of descriptors to measure emotional expressivity in different animal species (Wemelsfelder, 2007; Wemelsfelder and Mullan, 2014). In this assessment, the trained observer integrates multiple quantitative behavioral responses to describe the animal’s emotional state qualitatively. QBA has been used to assess the dogs in the shelter and home environments (Walker et al., 2016; Arena et al., 2019). Most recently, a fixed list of qualitative descriptive terms containing both positive (e.g., playful, curious, relaxed, tranquil) and negative (e.g., bored, apathetic, fearful, wary) connotations has been developed as a complementary assessment tool to evaluate the welfare of dogs in a shelter environment (Arena et al., 2019).

### Noninvasive Physiological and Endocrine Measures

There exists a close relationship between emotions and physiology, more particularly the autonomic nervous system (ANS) and the hypothalamic-pituitary-adrenal (HPA) axis activity (Sander and Scherer, 2014). Therefore, biomarkers play an important role and serve as a proxy when assessing emotion and positive well-being in animals (e.g.,von Borell et al., 2007; Schmied et al., 2008, 2010; Reefmann et al., 2009a, b; Coulon et al., 2013, 2015; Briefer et al., 2015; Kowalik et al., 2017). Heart rate (HR) (Csoltova et al., 2017), heart rate variability (HRV) (Bergamasco et al., 2010; Katayama et al., 2016; Travain et al., 2016), surface temperature (Travain et al., 2015, 2016; Csoltova et al., 2017), oxytocin (Odendaal and Meintjes, 2003; Mitsui et al., 2011; Rehn et al., 2014; Nagasawa et al., 2015), vasopressin (Hydbring-Sandberg et al., 2004; MacLean et al., 2017a, b, 2018; Pirrone et al., 2019), cortisol (Hennessy et al., 1998, 2006; Coppola et al., 2006; Bergamasco et al., 2010; Shiverdecker et al., 2013), and alpha-amylase (Contreras-Aguilar et al., 2017; Hong et al., 2019) have been implemented and showed potential usefulness in indirect and noninvasive assessment of positive-emotion in dogs.

#### Heart Rate

Monitoring HR responses has been utilized as an effective way for assessing the sympathetic branch activity of ANS activation in both animal and human studies (e.g., Beerda et al., 1998; Watkins et al., 1998; Loijens et al., 2002; Cyr et al., 2009; Csoltova et al., 2017). Itis widely accepted that measurements of HR are valid indicators of ANS activity and thus arousal and stress response (Ulrich-Lai and Herman, 2009). Although HR has been reported as a reliable indicator of arousal during behavioral tests, it is suggested that it cannot be used as an indicator of emotional valence (Beerda et al., 1998; Lensen et al., 2017).

Besides emotions, HR may be affected by different factors, for example, physical activity and temperature (Hales and Dampney, 1975; Maros et al., 2008). In dogs, it has been found that walking increased HR, which decreased during lying, although no differences in HR were found between sitting and standing (Maros et al., 2008).

Positive human-dog tactile contact has been shown to have an attenuating effect on cardiovascular responses of the dog (Anderson and Gantt, 1966; Lynch and McCarthy, 1967; McGreevy et al., 2005; Handlin et al., 2011; Csoltova et al., 2017; McGowan et al., 2018). In addition, the sole presence of the owner during a threatening situation has proved to have a stress-buffering effect on dogs’ HR, thus providing evidence for the “safe haven” effect of the owner on a dog’s well-being during a stressful situation (Gácsi et al., 2013).

Besides, HR monitoring was implemented when testing the hedonic aspects of food in dogs. Eating food was associated with initial increased cardiac activity, with a gradual return to baseline levels. The heart rhythm was found to be affected by the palatability of the food, with the most profound increase and decrease observed in response to the most preferred food (Kostarczyk and Fonberg, 1982). Similarly, a significant increase in HR was also observed when the dogs were offered a tasty treat (Travain et al., 2016).

#### Heart-Rate Variability

HRV has been shown to be an effective tool to measure the sympathetic and parasympathetic balance of the ANS (van Ravenswaaij-Arts, 1993; Thayer et al., 2010). HRV reflects variance in time intervals in successive heartbeats, indicating the organism’s capacity to regulate internal and external demands (van Ravenswaaij-Arts, 1993; Jarczok et al., 2015; Mccraty and Shaffer, 2015; Shaffer and Ginsberg, 2017).

There have been three approaches commonly utilized to monitor HRV (van Ravenswaaij-Arts, 1993; Electrophysiology Task Force of the European Society of Cardiology the North American Society of Pacing Electrophysiology, 1996; Shaffer and Ginsberg, 2017; Young and Benton, 2018; Table 1):

**TABLE 1:** Description of HRV measures (adapted from Electrophysiology Task Force of the European Society of Cardiology the North American Society of Pacing Electrophysiology, 1996; Shaffer and Ginsberg, 2017).

		Parameter	Unit	Description
Heart-rate variability	Time-domain measures	mean RR	ms	The mean of RR intervals
		SDRR/SDNN	ms	The standard deviation of RR/NN intervals
		RMSSD	ms	Root mean square of successive RR interval differences
		NN50		Number of successive NN intervals that differ more than 50 ms
		pNN50	%	Percentage of successive NN intervals that differ by more than 50 ms
	Frequency-domain measures	ULF	ms^2^	The absolute power of the ultra-low-frequency band (≤0.003 Hz)
		VLF	ms^2^	The absolute power of the very-low-frequency band (frequency range 0.0033–0.04 Hz)
		LF	ms^2^	The absolute power of the low-frequency band (frequency range 0.04–0.15 Hz)
		LF	n.u.	The relative power of the low-frequency band (frequency range 0.04–0.15 Hz) in normalized units
		HF	ms^2^	The absolute power of the high-frequency band (frequency range 0.15–0.4 Hz)
		HF	n.u.	The relative power of the high-frequency band (frequency range 0.15–0.4 Hz) in normalized units
		LF/HF	ms^2^	The ration between LF and HF band powers
	Nonlinear measures	SD1	ms^2^	Poincaré plot representing the standard deviation perpendicular to the line of identity (the standard deviation of instantaneous beat-to-beat R-R interval variability) (Tulppo et al., 1996)
		SD2	ms^2^	Poincaré plot representing the standard deviation along the line of identity (the standard deviation of continuous long-term R-R interval variability) (Tulppo et al., 1996)
		ApEn		Approximate entropy, quantifying the regularity and complexity of a time series (Pincus et al., 1991)
		SampEn		Sample entropy estimates the regularity and complexity of a time series (Richman and Moorman, 2000)
		DFA α1		Detrended fluctuation analysis, reflecting short-term fluctuations (Peng et al., 1995; Ho et al., 1997)
		DFA α2		Detrended fluctuation analysis, reflecting long-term fluctuations (Peng et al., 1995; Ho et al., 1997)
		D_2_		Correlation dimension measures the minimum number of variables required to construct a model of system dynamics

*RR, inter-beat intervals between all successive heartbeats; NN, normal inter-beat intervals between all successive heartbeats (removed abnormal beats/artifacts) (Electrophysiology Task Force of the European Society of Cardiology the North American Society of Pacing Electrophysiology, 1996; Shaffer and Ginsberg, 2017; Drury et al., 2020); ms, milliseconds; %, percentage; ms^2^, meter per second squared; n.u., normalized units calculated; LF [n.u.] = LF [ms^2^] / (total power [ms^2^] – VLF [ms^2^]); HF [n.u.] = HF [ms^2^] / (total power [ms^2^] – VLF [ms^2^]); Hz, hertz.*

1. Time-domain measurements (linear analysis) record the variability of the successive heartbeats during the measured time periods. They include parameters such as mean RR, SDRR/SDNN, RMSSD, NN50, and pNN50 (Shaffer and Ginsberg, 2017; Drury et al., 2020).
2. Frequency-domain (linear analysis) measure the absolute or relative power (the signal energy) distribution to different frequency bands such as ULF, VLF, LF, and HF (Electrophysiology Task Force of the European Society of Cardiology the North American Society of Pacing Electrophysiology, 1996; Shaffer and Ginsberg, 2017; Drury et al., 2020).
3. Nonlinear measures estimate the unpredictability and irregularity of time series (Stein and Reddy, 2005; Shaffer and Ginsberg, 2017; Young and Benton, 2018). They include, for example, SD1, SD2, ApEn, SampEn, DFA α1, DFA α2, and D2 parameters.

Assessing canine emotional states by implementing HRV indices has been gaining research popularity in recent years (Bergamasco et al., 2010; Gácsi et al., 2013; Bowman et al., 2015; Katayama et al., 2016; Travain et al., 2016; Zupan et al., 2016; McGowan et al., 2018; Köster et al., 2019). It has been proposed that HRV parameters might be sensitive indicators of emotional valence (Katayama et al., 2016) and tend to be less affected by physical activity compared to HR (Maros et al., 2008).

Positive-emotion-eliciting stimuli, such as food, human-dog interaction, and listening to music, all resulted in changes of HRV parameters in dogs (Bergamasco et al., 2010; Bowman et al., 2015; Travain et al., 2016; Zupan et al., 2016; McGowan et al., 2018).

An increase in RMSSD, pNN50, and HF parameters has been reported as an indicator of a positive-emotional state in dogs after 15 min of physical contact with humans (McGowan et al., 2018).

Exposure to auditory stimuli in the form of classical music resulted, among others, in mean RR, STDRR, RMSSD, pNN50, SD1, SD2 increase, and LF/HF decrease, indicating parasympathetic nervous system dominance and stress-buffering effects of music on dogs in a stressful environment (Bowman et al., 2015; Köster et al., 2019).

Zupan et al. (2016) studied the effects of positive stimuli on cardiac responses in dogs using higher- versus lower-valued food and social reward (familiar and less familiar person). Positive stimuli resulted in an increase of HR and LF/HF ratio, implying sympathetic nervous system activation and a positive arousal state in dogs throughout testing. In contrast to the aforementioned studies, exposure to the positive stimuli resulted in RMSSD and HF decrease compared to the baseline. A similar decrease was recorded for the LF compared with the baseline. A decrease in HF was observed when dogs were offered more preferred food (meatball) compared to less preferred food (commercial kibbles). A decrease in HF and RMSSD was recorded during the reward phase when the dog was allowed to interact with a person or eat the food compared to the anticipation phase when the dogs could only see the rewards. Authors proposed that higher positive-emotional valence in dogs is associated with parasympathetic deactivation. In this regard, lower RMSSD and HF combined are indicators of higher valence in an already positive-emotional state.

Interestingly, a negative experience in the form of isolation in an unfamiliar environment also resulted in RMSSD decrease in dogs. On the other hand, a decrease in SDNN was recorded when the dogs were petted by their owners (Katayama et al., 2016).

In another study, decreased SDNN has been linked to elevated attention, recorded when the dogs focused on their favorite ball (Maros et al., 2008).

When investigating cardiac responses to food treats during appetitive and reward phases, the authors found an increased HR, although no changes in HRV parameters were recorded. A significant increase in SDNN was observed after the positive stimulation occurred, during the post-consumption phase (Travain et al., 2016).

#### Superficial Temperature

Both positive and negative emotions activate ANS responses, which subsequently lead to physiological changes associated with alterations of blood flow, leading to surface temperature fluctuations (Sinha et al., 1992; Collet et al., 1997; Chotard et al., 2018).

Implementation of infrared thermography (IRT) is an effective way to accurately quantify the smallest superficial temperature changes in response to environmental and psychological stimuli, both in humans (Pavlidis et al., 2000, 2002; Pollina et al., 2006; Goulart et al., 2019; Panasiti et al., 2019; Zhang et al., 2019) and animals (Mccafferty, 2007; McManus et al., 2016; Telkanranta et al., 2018). IRT is a noninvasive imaging technique that detects the infrared wavelengths emitted by all objects with a temperature above absolute zero (Vollmer and Möllmann, 2017). Thermographic cameras provide a tool to monitor sudden rises and decreases in superficial temperature by either real-time observations or ultrahigh-speed video or thermal image recordings of studied objects.

In general, areas without the interference of fur are used as regions of interest to detect heat changes. Eye (Stewart et al., 2008; Csoltova et al., 2017) and nose (Kuraoka and Nakamura, 2011; Proctor and Carder, 2016) temperatures are used most frequently, but other body parts, such as the ears (Riemer et al., 2016) have also been used as a region of interest when studying emotion-induced heat surface changes in animals.

Due to the recent advances in thermal imaging technology and its noninvasive noncontact nature, the use of portable infrared cameras in animal emotion assessment research has been gaining popularity (e.g., Nakayama et al., 2005; Stewart et al., 2005, 2007, 2008, 2011, 2017; Kuraoka and Nakamura, 2011; Valera et al., 2012; Bartolomé et al., 2013; Herborn et al., 2015; Proctor and Carder, 2015; Cannas et al., 2018; Chotard et al., 2018; Hussein, 2018; Redaelli et al., 2019).

In dogs, there has been a growing interest in utilizing IRT to investigate negative emotion-induced surface heat increase associated mainly with stress, fear-based aggression, and separation anxiety, both in clinical and home settings (Travain et al., 2015; Riemer et al., 2016; Csoltova et al., 2017; Rigterink et al., 2018).

The affiliative behavior of the owner during a veterinary examination was found to have an attenuating effect on maximal ocular surface temperature compared to the no-interaction condition, reflecting possible parasympathetic activation (Csoltova et al., 2017).

The reappearance of both familiar and unfamiliar persons after a brief separation was sufficient to increase dogs’ outer ear temperature (Riemer et al., 2016).

The increase of maximal eye temperature was also recorded when dogs were anticipating and eating treats offered by owners (Travain et al., 2016).

From the research carried out so far, it is possible to conclude that IRT is a useful method to assess arousal intensity (Csoltova et al., 2017), but there has been some debate about whether IRT can distinguish emotional valence (Travain et al., 2015, 2016).

#### Oxytocin

Oxytocin is a neuropeptide and biomarker that is associated with a positive-emotional state in dogs (Mitsui et al., 2011).

Besides, previous studies indicate that in dogs, oxytocin may regulate a dog’s social behavior and attachment toward the owner (Beetz et al., 2012; Romero et al., 2014; MacLean and Hare, 2015; Nagasawa et al., 2015; Buttner, 2016; Kis et al., 2017; MacLean et al., 2017a). It seems that the sole reappearance of a familiar person, following separation, is sufficient to increase dogs’ oxytocin levels, but tactile and verbal contact is required for the oxytocin levels to remain continuously elevated (Rehn et al., 2014). Another study assessing the effect of positive human-dog interaction, found increased concentrations of biomarkers such as b-endorphin, oxytocin, prolactin, β-phenylethylamine, and dopamine in both humans and dogs (Odendaal and Meintjes, 2003). Similarly, other studies have supported the association between positive human-dog physical interaction and an increase in oxytocin in dogs (Handlin et al., 2011; MacLean et al., 2017a; Ogi et al., 2020).

Feeding and food-associated stimuli were equally found to increase oxytocin levels in dogs (Uvnäs−Moberg et al., 1985).

Moreover, the administration of exogenous oxytocin has been shown to have a pronounced effect on dogs’ cognition and behavior. Intranasal oxytocin administration increased the positive expectation of the dogs in the cognitive bias test (Kis et al., 2015), reduced separation anxiety (Thielke and Udell, 2017), and enhanced performance in a cognitive task (Oliva et al., 2015). Likewise, intranasal oxytocin administration increased dogs’ play motivation and intra- and interspecific social play behavior (Romero et al., 2014). Further, intranasally administered oxytocin has been shown to affect cardiac activity by decreasing HR and increasing HRV in dogs (Kis et al., 2014).

Urinary and, most recently, salivary oxytocin sampling has been validated as noninvasive approaches for quantifying oxytocin levels in dogs (Mitsui et al., 2011; MacLean et al., 2018; Powell et al., 2019; Schaebs et al., 2019; Wang et al., 2019).

However, as reported by Rault et al. (2017), it is still too early to consider oxytocin a potential indicator of positive well-being and/or welfare, given the discrepancies in the methodologies used to measure oxytocin in domesticated animals.

#### Vasopressin

Arginine vasopressin (AVP) is a neuropeptide closely related to oxytocin (Baribeau and Anagnostou, 2015). Both biomarkers play an important role in regulating mammals’ social and affiliative behavior (including pair-bonding and maternal behavior), social cognition, social stress and anxiety, and social aggression (Kendrick et al., 1997; Carter, 1998; Everts and Koolhaas, 1999; Goodson and Bass, 2001; Young and Wang, 2004; Donaldson and Young, 2008; Heinrichs and Domes, 2008; Veenema et al., 2010; Bisagno and Lud Cadet, 2014; Baribeau and Anagnostou, 2015).

In dogs, vasopressin increase has been associated with acute stress responses (Hydbring-Sandberg et al., 2004) and was also reported in dogs with separation anxiety (Pirrone et al., 2019). Further, lower free, but higher total plasma AVP has been found in aggressive dogs (MacLean et al., 2017b).

On the other hand, positive affiliative human-dog interactions, such as physical contact play, licking, lying supine have been associated with oxytocin increases, and AVP decreases (MacLean et al., 2017a).

Apart from blood sampling, salivary AVP has been validated as a potential noninvasive biomarker in dogs (MacLean et al., 2017b, 2018).

#### Cortisol

Increased cortisol levels are associated with HPA axis activation (Romero and Butler, 2007). Testing salivary cortisol levels in dogs has become a well-established method for measuring stress response and its impact on dogs’ well-being in several settings (Beerda et al., 1996, 1998; Dreschel and Granger, 2005; Haubenhofer and Kirchengast, 2006; Hekman et al., 2012; Glenk et al., 2014; Ng et al., 2014). Differences in the intensity and nature of a given stressor may impact endocrine responses, thus affecting cortisol release. A time frame from 10 to 30 min is required in order to detect a significant rise in cortisol concentrations in saliva after the onset of an acute stressor (Vincent and Michell, 1992; Beerda et al., 1998). To avoid the impact of handling on the measured cortisol concentration, the saliva sample collection time should not exceed 4 min (Kobelt et al., 2003).

There exists evidence suggesting an inverse relationship between cortisol and positive-emotional state in humans (Lindfors and Lundberg, 2002; Lai et al., 2005). Decreased cortisol levels have been implemented as an indicator of the stress-buffering effect of stimuli in several dog welfare studies. Such positive effects have been observed in human-dog interaction and listening to music, as dogs’ cortisol levels showed a decrease when studied in an animal shelter (Hennessy et al., 1998, 2006; Coppola et al., 2006; Bergamasco et al., 2010; Shiverdecker et al., 2013) and veterinary environments (Csoltova et al., 2017).

#### Alpha-Amylase

As with cortisol, measuring decreased levels of alpha-amylase, a crucial salivary enzyme (Nater and Rohleder, 2009), is another noninvasive approach to test sympathetic nervous system deactivation. Salivary alpha-amylase increase in response to both acute and chronic stressors has been documented in different animal species (Fuentes et al., 2011; Behringer et al., 2012; Fuentes-Rubio et al., 2016; Contreras-Aguilar et al., 2018), including dogs (Contreras-Aguilar et al., 2017; Hong et al., 2019). However, other studies emphasize the involvement of the parasympathetic nervous system branch in alpha-amylase secretion (Asking and Proctor, 1989; Nater and Rohleder, 2009; Bosch et al., 2011).

### Noninvasive Neurobiological Methods

Increased tendencies to use neurobiological approaches to investigate the canine brain, social intelligence, and emotion have been observed in recent years. Functional magnetic resonance imaging (fMRI) and functional near-infrared spectroscopy (fNIRS) have been implemented to noninvasively study dog brain responses to both positive and negative emotion stimuli (e.g., Andics et al., 2014; Cook et al., 2014, 2016, 2018; Gygax et al., 2015; Berns and Cook, 2016).

#### Functional Magnetic Resonance Imaging

fMRI is a neuroimaging tool used to measure and quantify brain activity indirectly by detecting dynamic changes in brain tissue oxygenation (Chen and Glover, 2015). Research studies mostly use the fMRI blood oxygen level-dependent imaging (BOLD) method, which reflects changes in blood oxygenation, flow, and volume in response to neural activity (Heeger and Ress, 2002; Glover, 2011; Devlin, 2016).

Thus far, studies using fMRI have focused on identifying neural responses to reward preference, temperament, face, odor, and vocal processing in awakened dogs (Andics et al., 2014; Cook et al., 2014, 2016, 2018; Berns et al., 2015; Dilks et al., 2015).

When studying the neural bases of preferences between social and food reward, researchers found that individual differences in mean ventral caudate activation in response to praise versus food predicted the subsequent behavioral choice between interaction with the owner (verbal praise) and food reward (eat a treat). Interestingly, 13 out of 15 tested dogs showed a preference for praise over food (Cook et al., 2016).

In another study, aggressive temperament was found to positively correlate with amygdala activation in dogs, while watching their caregiver give food to a fake dog. The authors interpret this emotion as a complex, jealousy-like phenomenon (Cook et al., 2018).

Another fMRI study showed that dogs are sensitive to human and dog auditory stimuli of different positive and negative emotional valence. Emotion-eliciting sound signals activated similarly located non-primary auditory areas both in dog and human brains. Besides, more pronounced neural activation was recorded to positive sound stimuli in both humans and dogs (Andics et al., 2014).

Olfactory neuroimaging study with dogs provided evidence for scent discrimination and positive associations with different odors, reflected in the maximal activation of the caudate nucleus to the odor of a familiar human in contrast to the odor of a familiar dog (Berns et al., 2015).

Besides, there is evidence suggesting a relationship between certain canine temperament traits and caudate nucleus activation. Twelve dogs were presented with 15 reward and 15 non-reward signals delivered by a familiar human, an unfamiliar human, or screen projected computer-generated signals during fMRI sessions. Results revealed higher caudate activation for reward versus non-reward signals. Dogs scoring lower on the Canine Behavioral Assessment and Research Questionnaire (C-BARQ) aggressivity trait showed higher caudate activation to the reward than non-reward signals when delivered by a familiar human. On the contrary, in dogs scoring higher on the aggressivity trait, higher differential responses to the reward versus non-reward signals presented by the unfamiliar human and computer projected signals were observed (Cook et al., 2014).

#### Functional Near-Infrared Spectroscopy

fNIRS is another neuroimaging method and portable equipment used for noninvasive functional mapping of brain activity by using near-infrared detectors to measure cortical concentrations of oxygenation/deoxygenation and hemodynamic changes by absorbing infrared light by hemoglobin (Tamura et al., 1997; Ferrari and Quaresima, 2012).

fNIRS has been used in canine research to assess positive-emotional states during verbal and tactile human interactions. The results revealed that the cortical hemodynamic reaction measured by fNIRS may be a useful indicator of the emotional valence, while measured behavioral responses were shown as useful indicators of arousal level in dogs. The consistency of the hemodynamic frontal cortical reaction throughout the test, in addition to changes in behavioral responses with repetition, indicates that the valence of the stimuli remained the same while the arousal of the dogs decreased as dogs habituated to the repetitions (Gygax et al., 2015).

## Possible Future Direction in Dog Positive-Emotion Assessment

### Psychophysiology

Historically, psychophysiology has been interested in studying the impact of emotional processing on physiological functions (Davidson, 2003). With the rapid development of new technologies in the pet care industry, implementing complementary psychophysiology biosensors could shed additional light on our understanding of canine positive-emotions.

#### Pupil Dilation

Pupil dilation is associated with the interactions of sympathetic and parasympathetic innervations of the ANS in the iris muscle. According to findings in human research, pupil response is a sensitive indicator of emotional arousal, irrespective of the hedonic valence of the stimuli. Pupil dilation was observed during emotionally engaging visual (Bradley et al., 2008) and auditory stimuli (Partala and Surakka, 2003), implying increased sympathetic activation during both pleasant and unpleasant stimuli presentation (Bradley et al., 2008).

It would certainly be interesting to investigate the role of the emotional valence of different types of rewarding stimuli (such as visual, gustatory, olfactory, or auditory stimuli) on pupil dilation in dogs.

#### Galvanic Skin Response

The galvanic skin response (GSR), also known as the skin conductance response or electrodermal response, is based on continuous autonomic variation in the electrical properties of the skin and results from sympathetic activation when either external or internal stimuli occur that are physiologically arousing (Lykken and Venables, 1971). GSR has been closely linked to autonomic emotional and cognitive processing and reflects sympathetic arousal. It is the only autonomic psychophysiological variable not affected by parasympathetic activity. This method can be implemented in an objective assessment of emotional states and attentional processing (Braithwaite et al., 2013).

GSR was sensitive to stimulus valence in humans presented with pleasant and unpleasant food and nonfood pictures. The pleasant pictures were associated with a decrease in skin conductance, while the negative pictures evoked sweating and increased skin conductance. This indicates the potential effectiveness of GSR in a valence assessment of emotional stimuli (Kuoppa et al., 2016).

In addition, a close relationship between GSR and other psychophysiology measures was found. An accuracy rate of 80.2% was observed between HRV and GSR signals in association with emotion assessment (Lee et al., 2005). High covariation between pupillary dilation and GSR was recorded in reaction to positive and negative picture viewing, suggesting sympathetic nervous system activation in humans (Bradley et al., 2008).

#### Respiration Rate

Autonomic respiration is regulated by metabolic requirements but can shift in response to changes associated with different emotions, such as sadness, happiness, anxiety, or fear (Homma and Masaoka, 2008). Research on emotion-respiration relationships has largely focused on measuring respiration rate, amplitude, volume, and respiratory cycle (Boiten et al., 1994). In human literature, a study suggests that different emotional states can be differentiated according to the type of breathing pattern (Philippot et al., 2002). In farm animals, the respiration rate seems to be a useful indicator of emotional arousal (Briefer et al., 2015), and possibly also of emotional valence (Reefmann et al., 2009a). Therefore, measuring the respiration rate can shed additional light on emotion processing in dogs.

#### Immunological Indicators

Many studies on humans found a distinct enhancement effect of positive-emotions on the immune system (Dillon et al., 1986; Pressman and Black, 2012; Stellar et al., 2015). In dogs, only the impact of negative stress on the immune reaction has been studied so far. According to the findings, acute stress affects the immune system by inducing overall peripheral leukocytosis (Beerda et al., 1997). Salivary immunoglobulin A (sIgA) has been proposed as another potentially useful bioindicator of acute and chronic stress in dogs (Skandakumar et al., 1995; Kikkawa et al., 2003).

Association between canine immune system enhancement and positive-emotional state is another research area that requires more attention. Salivary immunological indicators could find their potential usefulness, especially in the assessment of dogs’ longer-term emotional states and positive well-being.

### Affective Computing in Positive-Emotion Assessment

Both machine learning and deep learning have promising potential in animal behavior, communication, emotion, and welfare research (Valletta et al., 2017; Hantke et al., 2018; Norouzzadeh et al., 2018; Pereira et al., 2019). Machine learning has been used for automatically computing interpretable, quantitative measures and classification of behaviors and activities within complex data sets (Kabra et al., 2013; Dell et al., 2014). Machine learning has been widely applied especially to animal movement and location data analyses (Wang, 2019). In human research, machine learning techniques have already been implemented to recognize emotional states by studying facial expressions (Michel and Kaliouby, 2003), speech (Shami and Verhelst, 2007), body movements and gestures (Castellano et al., 2007), and also physiological responses such as cardiac activity, galvanic skin response, respiration rate, and skin temperature (Shi et al., 2010; Domínguez-Jiménez et al., 2020). Also, infrared thermography data are showing promising usefulness in automatic emotion recognition (Boccanfuso et al., 2016).

Deep learning, a subfield of machine learning, goes further by using a deep neural network to learn from and process raw data (Goodfellow et al., 2015). It has been gaining popularity in human emotion recognition studies (Giannopoulos et al., 2018; Jain et al., 2018; Hassan et al., 2019).

Implementation of affective computing in positive-emotion assessment might be particularly useful where sorting and classifying a large data set are required or where multimodal data integration is necessary to further enhance emotion recognition (Caridakis et al., 2007; Yin et al., 2017). Affective computing might provide solutions in accurate automatic detection of the small subtleties related to different emotional states by analyzing data from either a single biosignal input or by a combination of multiple physiological inputs. Affective computing might also provide solutions for the recognition of emotional states with similar biosignal inputs by analyzing data in a time series (Quesnel et al., 2018). Furthermore, affective computing considerably decreases the time of manual data and video analyses.

In canine emotion research, machine learning has thus far shown promising results in individual, context, and emotion (both valence and intensity) recognition of dog barks (Molnár et al., 2008; Hantke et al., 2018).

Overall, both machine and deep learning offer great potential in automatic emotion detection and recognition (Yin et al., 2017).

## The Impact of Human-Dog Relationship on Dog Emotion

There is an increasing amount of evidence suggesting that the unique characteristics of the human-dog relationship, owner’s personality, emotional state, or behavior have been shown to affect the behavior, cognition, emotion processing, and expression of pet dogs (Prato-Previde et al., 2008; Handlin et al., 2012; Merola et al., 2012; Gácsi et al., 2013; Konok et al., 2015a; Cimarelli et al., 2016; Schöberl et al., 2016; Huber et al., 2017; Dodman et al., 2018; Gobbo and Zupan, 2020; Salamon et al., 2020).

The relationship between owner and dog resembles that of the parent (primary caretaker) and child (Bowlby, 2005). The dog perceives the owner as a secure base from which to explore the world (Topál et al., 1998). Pet owners differ in their attachment style to dogs, and these differences in behavior and emotion toward dogs impact their behavior and emotion toward their dog, subsequently affecting the dogs’ interaction with the environment. The owner’s attachment and caregiving style have been shown to impact dogs’ responses toward stress-inducing stimuli. The dogs of secure owners showed the most independent and confident behavior when they were exposed to environmental stressors compared with dogs of anxious and avoidant owners (Rehn et al., 2017). Moreover, dogs of owners with insecure attachments had a higher tendency to develop separation anxiety (Konok et al., 2015a).

Some studies have found a link between the owner’s personality and dogs’ behavioral and physiological responses. Owners scoring high in neuroticism and agreeableness had dogs with lower cortisol levels measured in the Strange Situation Test (Schöberl et al., 2016). In addition, dogs pay close attention to owners’ responses and act accordingly when facing novel stimuli (Merola et al., 2012; Salamon et al., 2020).

Further, research studies have shown that dogs tend to synchronize their emotional states with their owner (Sümegi et al., 2014; Turcsán et al., 2015; Sundman et al., 2019). Emotional contagion is an immediate synchronization of the emotional state between the subject and an object (Preston and de Waal, 2002; Nakahashi and Ohtsuki, 2018). Emotional contagion from the owner to the dog was recorded by the assessment of the cardiac responses during the Trier social stress test (TSST). Synchronization of the RMSSD levels between the owner and the dog during a stressful condition was found to positively correlate with the duration of the ownership (Katayama et al., 2019). Oxytocin-level correlation was recorded between the owner and the dog during a short-term interaction (Handlin et al., 2012). Owner-dog synchronization of cortisol levels was confirmed during dog agility competition (Jones and Josephs, 2006; Buttner et al., 2015). Furthermore, a recent study showed evidence of long-term synchronization of stress levels between owners and dogs (Sundman et al., 2019). Research on interspecies human-dog chemo-signaling revealed that dogs’ behavioral and heart responses were affected by human body odor changes, induced by different emotional states such as happiness and fear (D’Aniello et al., 2018).

## Conclusion and Further Directions

Our main goal was to review and summarize the current advancement and scattered and dispersed research on positive-emotion in dogs. As previously discussed, prior research has focused on behavioral, cognitive, physiological, endocrine, and neurobiological approaches in dogs’ positive-emotion assessment.

Behavioral indicators have been shown to be contradictory and highly context-dependent. Therefore, environmental conditions need to be taken into consideration when interpreting the results. Another major issue with behavioral indicators is the high intra- and interindividual variability in responses (Beerda et al., 1997; Bell et al., 2009; Biro and Adriaenssens, 2013; Goold and Newberry, 2017). One possible solution to these issues could be the implementation of artificial intelligence in behavior analyses. Machine learning and deep learning algorithms could provide useful solutions in detecting subtle differences in behavioral responses caused by different valence and try to find response patterns to a specific positive-emotion eliciting stimulus. But for the moment, behavioral parameters should be used as complementary indicators of emotional valence and supported by other measurements.

Research findings on cerebral asymmetry have identified diverse lateral biases toward qualitatively different emotion stimuli, suggesting the relevance of this asymmetry to animal well-being and welfare research (Rogers, 2010). However, further research on brain lateralization is necessary to deepen our understanding of positive-emotion and emotional information perception, processing, and response in dogs.

Implementing cognitive bias testing is another promising means to assess the emotional responses and overall well-being of a dog (Burman et al., 2011; Kis et al., 2015; Wells et al., 2017). Therefore, more research studies are required in this area. However, the role of temperament traits as a predictor of cognitive bias remains to be further explored (Barnard et al., 2018).

More and more evidence suggests that QBA is a reliable, dynamic qualitative method for positive-emotion and welfare assessment (Stockman et al., 2011; Fleming et al., 2016; Minero et al., 2016, 2018; Collins et al., 2018; Patel et al., 2019). This tool seems to be particularly beneficial in assessing the overall well-being of dogs, for example, in a shelter environment (Walker et al., 2016; Arena et al., 2019).

Certain physiological (e.g., heart rate and superficial temperature) and neurobiological (e.g., amygdala activation) measures are good indicators of arousal; however, they have also been shown to correlate with more than one type of emotional state (Beebe-Center and Stevens, 1937; Kostarczyk and Fonberg, 1982; Garavan et al., 2001; Hamann et al., 2002; Bonnet et al., 2015; Proctor and Carder, 2016; Travain et al., 2016). Again, applying artificial intelligence in data analysis to find specific patterns of responses or changes in time series might prove useful in the identification of qualitative and quantitative differences in positive-emotion processing and responses.

HRV markers have been found to be sensitive to both valence and intensity of emotion (Katayama et al., 2016; Zupan et al., 2016). These promising findings need to be further explored by implementing additional HRV parameters to measure valence and arousal aspects of positive-emotion-eliciting stimuli under well-controlled conditions.

Some of the noninvasive salivary endocrine measures have been validated only recently, and more research is needed in the area. Nevertheless, the common methodological issues in endocrine measures have been identified as subject-related (intra- and interindividual variability), experiment-related (lack of standardization in methodology, impact of sampling time, owner presence, and data interpretation), and context-related (both the impact of testing and regular living environment). Standardized methodology and well-controlled experiments are required to validate endocrine measures as reliable indicators of positive well-being in animals (Cobb et al., 2016; Rault et al., 2017; Chmelíková et al., 2020).

Research evidence shows that both fMRI and fNIRS might be very useful tools in the systematic exploration of positive-emotion processing in dogs. fNIRS and fMRI are comparative methods sensitive to similar physiologic changes. While implementing neuroscientific research into dogs’ positive assessment can enhance our knowledge, the fMRI is technically demanding and requires pre-trained animals, which limits studies to small sample sizes. On the other hand, fNIRS is portable, with higher tolerances to motion, and cost-effective; and it requires no restraint of animals. The disadvantage of fNIRS over fMRI is a limited spatial resolution (Scarapicchia et al., 2017).

Furthermore, we have proposed additional psychophysiology approaches which could be complementary to the existing methods in the assessment of both short-term emotional responses as well as longer-term emotional states. Moreover, we would like to stress again the great potential and importance of affective computing in animal emotion research, especially in recognizing subtle differences among emotional responses or by detecting specific patterns in time series of single biosignals or by combining multimodal input data (Caridakis et al., 2007; Yin et al., 2017; Quesnel et al., 2018). For example, deep learning might be particularly helpful in developing a newer methodology for fast detection of regions of interest and accurate, reliable data extraction and analyses in infrared imaging (Marzec et al., 2016; Sonkusare et al., 2019).

Overall, it is too early to conclude whether a certain method or technique in positive-emotion research on dogs is more suitable than others. Prevalent methodological issues and the chronic problem of small sample sizes show that there is a huge need for well-controlled studies with larger numbers of participants. Some approaches show more promising results than others, but the great amount of ambiguous and contrasting findings points toward certain issues that need to be addressed before proceeding further:

a. The relevance of dog temperament in emotion perception, regulation, and responses. Research findings indicate the importance of temperament in dogs’ emotion processing and responses (e.g., Cook et al., 2014, 2018; Barnard et al., 2018). Thus, the role of temperament in dog emotion assessment deserves to be investigated further. Implementing validated and reliable dog temperament questionnaires (e.g., PANAS, C-BARQ, MCPQ-R, VIDOPET) (Sheppard and Mills, 2002; Hsu and Serpell, 2003; Ley et al., 2009; Rayment et al., 2016; Turcsán et al., 2018; Savalli et al., 2019) could potentially reduce variability in studies (Manteca and Deag, 1993) and shed additional light on inter-individual differences in dogs’ emotion processing and responses.
b. Establishing the reward value prior to testing. Positive-emotion eliciting stimuli used in previous studies are mostly pleasure-related (e.g., hedonic taste, pleasurable touch). However, not all food is automatically *liked* by, and thus pleasure-eliciting for all dogs. The liking aspects depend both on individual preferences and levels of hunger, satiation, or satiety. Despite the fact that owner-dog physical interaction is generally regarded as a positive social reward, not all human interaction might present a positive experience. For example, petting dogs in certain areas might be an unpleasant experience for a dog (Kuhne et al., 2014a). Therefore, close attention needs to be paid to the real value of the reward to a dog. Besides, the arousal aspect of the stimulus should be taken into account as well, since it might affect the response strength. The impact of the testing environment might further complicate the issue, since a novel environment might present a stressor to a dog and thus possibly reduce the hedonic valuation of rewards.
c. Dissecting *wanting* and *liking* components of reward (Berridge et al., 2009). When measuring responses to pleasure eliciting stimuli, researchers should focus separately on each phase of the pleasure cycle (Figure 1). As we have previously discussed, distinct reward-related motivational, emotional, and cognitive processes are activated, each with separable neurobiological mechanisms during appetitive, consummatory, and satiety phase (Berridge et al., 2009; Berridge and Kringelbach, 2011). Furthermore, reward delay during the anticipatory phase may lead to frustration (Amsel, 1958).
d. Controlling the impact of dogs’ social cognition, attachment, and emotion contagion. The unique psychological characteristics of the owner-dog relationship might significantly impact the measured emotion responses in dogs. Owner-dog relationship or attachment style questionnaires (e.g., MDORS, PAQ) (Dwyer et al., 2006; Zilcha-Mano et al., 2011) could be implemented when dogs are being tested in the presence of the owner, or when owner-dog interaction is used to induce positive-emotion in dogs. Besides, as dogs pay close attention to human emotional signals and cues and act accordingly, this must be taken into consideration during testing conditions as well (Merola et al., 2012; Salamon et al., 2020).

As the research findings show, no single valid method currently exists to reliably assess positive-emotion in dogs. Therefore, great caution is advised when interpreting behavioral, physiological, or endocrine indicators alone (de Vere and Kuczaj, 2016). Multiple noninvasive approaches, combining behavioral, noninvasive physiological, endocrine, and neural measures, need to be implemented to get a rigorous and reliable assessment of qualitative and quantitative differences of emotions in dogs (Paul et al., 2005; Boissy et al., 2007; de Vere and Kuczaj, 2016).

Overall, this review indicates that the research field concerning canine positive-emotion remains largely unexplored, offering researchers opportunities for discoveries that would deepen our knowledge of positive-emotion perception, processing, and recognition, with possible implications for both research and practice to improve the positive well-being and welfare of our companion animals.

## Author Contributions

EC reviewed the literature and wrote the manuscript. EM reviewed and approved the final manuscript. Both authors contributed to the article and approved the submitted version.

## Conflict of Interest

EC and EM were employed by the company Diana Pet Food.

## Footnotes

1 Even though *temperament* and *personality* are often used interchangeably or are not clearly distinguished in animal studies, we adopt the view from human psychology that *personality* is considered a broader concept encompassing individual characteristics that represent a unique consistent pattern of feeling, thinking, and behaving (Cervone and Pervin, 2015).

2 For the purposes of this paper, *well-being* is defined as the animal’s complex subjective internal state affected by the animal’s perception, cognition, emotion, motivation, and the manifestation of its mental and somatic perception and interpretation of the internal and external stimuli (Clark et al., 1997). The term *welfare* is considered broader, encompassing the overall state of animal’s health and physical, social, and mental well-being (adapted from Halverson, 2001). The exception is when we are discussing the work of others who use a different convention.
